# Isolation, identification, antibiotic resistance profile and molecular analysis of *Ornithobacterium rhinotracheal* isolates from turkeys

**DOI:** 10.1002/vms3.1490

**Published:** 2024-06-05

**Authors:** Sedigheh Yusefinejad, Darioush Gharibi, Mohammad Khosravi, Mansour Mayahi, Masoud Reza Seyfi Abad Shapouri

**Affiliations:** ^1^ Department of Pathobiology, Faculty of Veterinary Medicine Shahid Chamran University of Ahvaz Ahvaz Iran; ^2^ Department of Pathobiology Faculty of Veterinary Medicine Shahid Chamran University of Ahvaz Ahvaz Iran; ^3^ Department of Clinical Sciences Faculty of Veterinary Medicine Shahid Chamran University of Ahvaz Ahvaz Iran

**Keywords:** antibiotic resistance, ERIC PCR, *Ornithobacterium rhinotracheal*, RAPD PCR, turkey, virulence genes

## Abstract

**Background:**

*Ornithobacterium rhinotracheal* (ORT) infects numerous birds, particularly chickens and turkeys. ORT is an emerging bacterial pathogen of global concern in the poultry industry. As ORT is rapidly spreading throughout commercial poultry, it requires intensive studies of its epidemiology, diagnostic procedures, molecular typing, virulence genes and antimicrobial resistance.

**Objectives:**

The present study was conducted in isolation and identification of ORT from slaughtered turkeys.

**Methods:**

Cleft palate swabs of 200 were collected from slaughtered turkeys and cultured on blood agar. ORT was characterized using biochemical tests and PCR targeting the ORT 16S rRNA gene. Virulence genes of isolates were determined targeting adenylate kinase (*adk)*, *copA* and virulence‐associated protein D (*vapD*) genes. Additionally, diversity of ORT isolates was performed by enterobacterial repetitive intergenic consensus (ERIC) and RAPD PCR. Disk diffusion was used to determine the antibiotic sensitivity of the isolates.

**Results:**

ORT was identified in 23 (11.5%) samples using both the biochemical tests and PCR. The result of detecting virulence genes showed that all the isolates (23: 100%) had the *adk* gene, whereas two (8.7%) isolates had the *copA* gene, and seven (30.43%) isolates had the *vapD* gene. Molecular typing of isolates revealed 21 different patterns by RAPD PCR assay using M13 primer and 20 distinct patterns by ERIC PCR test. Both ERIC and RAPD PCR were distinctive methods for investigating the genetic diversity of *ORT* isolates. The antibiotic resistance test showed that 18 (78.26%) isolates were resistant to gentamicin, amikacin, cefazolin, streptomycin and penicillin. All isolates (100%) were resistant to cloxacillin and fosfomycin.

**Conclusions:**

This study showed the prevalence of ORT in turkey and high resistance of this bacterium to many common veterinary antibiotics. Moreover, both ERIC and RAPD PCR are distinctive methods for investigating the genetic diversity of ORT isolates. These data may help monitor antibiotic resistance and typing of ORT in epidemiological studies and serve as the foundation for designing region‐specific vaccines for future use.

## Graphical abstract

*Ornithobacterium rhinotracheal* (ORT) was identified (11.5%) in turkey.

The isolates had the *adk*, *copA* and *vapD* gene.

Twenty‐one different patterns were identified in RAPD PCR.

Twenty distinct patterns of isolates were determined by ERIC PCR test.

Most isolates were resistant to gentamicin, amikacin, cefazolin and cloxacillin.

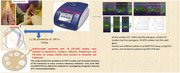

## 1 INTRODUCTION

*Ornithobacterium rhinotracheal* (ORT) is a gram‐negative, rod‐shaped, pleomorphic, microaerophilic, non‐motile, non‐sporulating, catalase‐negative and oxidase‐positive bacterium that was first reported in domesticated poultry and wild birds (Amonsin et al., 1997). The ORT was first detected from respiratory disease syndrome in turkeys in the early 1990s in Germany (Charlton et al., 1993) and was named in South Africa in early 1994 (Vandamme et al., 1994). The genus *Ornithobacterium* belongs to the family *Weeksellaceae*. Besides the genus *Ornithobacterium*, there are two genera, *Riemerella* (*R. anatipestifer*) and *Coenonia* (*C. anatine*), which are the most significant bird pathogens, especially in geese and domestic ducks (Veiga et al., 2019). *Ornithobacterium* was first identified as a non‐haemolytic bacterium, but species with haemolytic activity have also been identified from commercial flocks of chickens and turkeys (Bordoloi et al., 2020; Tabatabai et al., 2010).

ORT is isolated from poultry flocks around the world, including Germany, USA, Israel, South Africa, France, the Netherlands, Hungary, Austria, Slovenia, Belgium, Italy, England, Ireland, Canada, Peru, India, Turkey, Thailand, Taiwan, Malaysia, Korea, China, Japan and Indonesia (DE OCA‐Jimenez et al., 2018). The first report of ORT infection in Iran was in 2000 from a flock of broiler chickens and laying hens with respiratory symptoms (Banani et al., 2000). ORT infection, also known as ornithobacteriosis, is a significant disease in commercial turkeys that often manifests as respiratory diseases and can lead to growth retardation, reduced feed and water intake, decreased egg production, and increased mortality. ORT is an emerging bacterial pathogen of global concern in the poultry industry (Ha et al., 2016; Smith et al., 2020; Thieme, Mühldorfer, et al., 2016). *Ornithobacterium* infection has been reported in commercial poultry and many other bird species. ORT is rapidly spreading throughout commercial poultry and non‐galliform birds. Wild birds are considered a source of infection for commercial poultry flocks (Alispahic et al., 2021).

Global poultry meat consumption has significantly increased in the last few decades. Consumer demands for fresh meat and poultry products with low fat and high protein have led to turkey meat ranking as the second most popular poultry meat around the world. In Iran, turkey production has the highest economic importance after chicken production (Hiscock et al., 2022; Kheiralipour et al., 2017). Considering the importance of ornitobacteriosis to the poultry industry, it should be monitored and included in national programmes for the prevention and control of avian respiratory diseases.

Pathogenesis severity in ORT is associated with environmental factors, biofilm formation and synergistic effects with other pathogens. A plasmid known as pOR1 is found in ORT. This plasmid contains a range of virulence genes, such as heavy metal resistance (*copA)*, biofilm production (virulence‐associated protein D [*vapD*]) and antibiotic resistance genes. The copA (multicopper oxidase domain‐containing protein) confers bacterial resistance to heavy metals such as cadmium, cobalt and zinc and is an H–K antiporter and copper‐exporting ATPase. The pOR1 plasmid may also contain *vapD*, which may be associated with biofilm production. This plasmid may also play a role in the emergence of resistance to antibiotics that are often used to treat ORT (Smith et al., 2020). ORT also had an adenylate kinase (*adk*) gene known as the virulence gene in ORT. *adk* is a housekeeping gene and plays an important role in cellular energy homeostasis and adenine nucleotide metabolism (Erfan & Marouf, 2019).

Because clinical signs and associated post‐mortem lesions with ORT infections are variable and nonspecific, isolation and specific laboratory methods are required for definitive diagnosis (Ellakany et al., 2019). Molecular methods are specific methods with high specificity and reliability in diagnosing *Ornithobacterium* infection, and often the 16S rRNA gene is used in the molecular diagnosis (Thieme, Mühldorfer, et al., 2016). The ERIC‐PCR (enterobacterial repetitive intergenic consensus) technique is a fast, simple, inexpensive, reliable and efficient tool for fingerprinting the ORT genome, which is used to differentiate bacterial strains isolated from different sources. Indeed, the high discriminatory power of isolates is a key advantage of this technique (Bilung et al., 2018; Igwaran & Okoh, 2020). RAPD‐PCR has the power to analye the phylogenetic relationship between strongly related species and can distinguish between strains in a species, and it is an excellent and valuable tool for epidemiologic surveys (Stefańska et al., 2022; Williams et al., 1990).

There is evidence of ORT acquiring antibiotic resistance easily, and sensitivity/resistance distinctions may vary according to the strain's phenotypic profile and geographic origin. Different therapeutic strategies for using antibiotics and indiscriminate use of them can lead to the creation of various antibiotic‐resistant strains. As an increasingly wide range of antimicrobial agents become ineffective against ORT, it confirms the hypothesis of continuous resistance transference among them, resulting in increased resistance for different drug classes (Bordoloi et al., 2020). It is therefore necessary to determine antibiotic sensitivity for effective ornithobacteriosis treatment.

As ORT is rapidly spreading throughout commercial poultry, it requires intensive studies of its epidemiology, diagnostic procedures, molecular typing, virulence genes and antimicrobial resistance. The poultry industry should include reporting and monitoring of ornithobacteriosis in a national programme aimed at preventing and controlling avian respiratory diseases, given its economic significance. To our knowledge, studies concerning virulence‐associated genes, antibiogram profile and genotyping of ORT from turkey in Iran are scarce. Therefore, the current study aimed to investigate these features in turkey ORT isolates to increase knowledge.

## 2 MATERIALS AND METHODS

### 2.1 Sample collection

Between January 2021 and May 2022, 200 samples of swabs were randomly collected from the cleft palate of slaughtered turkeys with and without respiratory symptoms. The samples were taken in the slaughterhouse of Khuzestan province, south‐western Iran. Swab samples were transferred in Cary Blair transport medium and cultured in less than 12 h.

### 2.2 Bacteriological examinations

Samples were cultured on a blood agar medium containing 5% horse blood and incubated in a candle jar with 7.5% CO_2_ and in humid at 37°C for 24–48 h. Colonies morphologically similar to ORT (fine, pinhead, round, grey, opaque, haemolytic and/or non‐haemolytic with a diameter of 1–3 mm) were selected. After purification, their initial identification was performed by gram stain and biochemical tests, including catalase, oxidase, nitrate reduction, motility, indole production, urease and fermentation of arabinose, sorbitol, glucose, lactose, maltose and sucrose according to laboratory instructions (Ellakany et al., 2019; Hafez, 2002).

### 2.3 Molecular analysis

DNA extraction: DNA of suspected isolates of ORT was extracted by the simple boiling method. Single colonies were suspended in microtubes containing 100 µL of sterile distilled water and heated at 100°C for 10 min then centrifuged for 2 min at 4000 rpm (Szabó et al., 2017). The supernatant was used as template DNA for molecular studies and stored in a freezer at −20°C until use.

#### 2.3.1 Identification of ORT by PCR

The ORT isolate was confirmed by specific PCR that amplifies a specific fragment (784 bp) of the 16S rRNA ORT gene as described by Hafez (2002). The sequence primer pairs are stated in Table 1. The PCR was performed in a final volume of 25 µL comprising: 12.5 µL of Master Mix (Amplicon), 1 µL of each primer (10 pmol/µL), 5 µL (500 ng) of template DNA and 5.5 µL of sterile distilled water. The thermal cycle condition is shown in Table 1. PCR products of 6 µL were electrophoresed (96 V for 1 h) in 1% agarose gel with 0.5 µg/mL safe stain, and DNA fragments were observed by UV transillumination and compared with a 100 bp DNA marker. Serotype A of ORT and distilled water were used as positive and negative controls, respectively.

**TABLE 1:** Sequences and cycling conditions of the 16S rRNA, virulence genes, enterobacterial repetitive intergenic consensus (ERIC) and RAPD PCR primers of *Ornithobacterium rhinotracheale* isolates.

Genes	Sequence (5 ′–3′)	Size	Secondary denaturation	Annealing	Extension	References
16S rRNA	F: GAGAATTAATTTACGGATTAAG R: TTCGCTTGGTCTCCGAAGAT	784 bp	94°C 30 s	53°C 40 s	72°C 40 s	Hafez (2002)
*adk*	F: GGCAGTGGAAAAGGAACTCA R:TCTAAACTTCCTTCGCCGTTT	502 bp	94°C 45 s	52°C 40 s	72°C 30 s	Erfan and Marouf (2019)
*copA*	F: CGACACTGCCGAAATAGTGCGC CopA R: AACGGTTAGCGTCGTTATCCGG	1039 bp	94°C 45 s	58 s 40 s	72°C 30 s	This study
*vapD*	F:ATGTACGCAATAGCATTTGACGCG R: GCTCTGATATCTCTTACAGACGCG	223 bp	94°C 45 s	56°C 40 s	72°C 30 s	This study
ERIC	(5′‐ATGTAAGCTCCTG GGGATTCAC‐3′)	Vary	94°C 45 s	40°C 180 s	72°C 240 s	Thachil et al. (2007)
M13	(5′‐TTAT GTA AAA CGA CGG CCA GT‐3)	Vary	92°C 60 s	35°C 30 s	72°C 90 s	Thachil et al. (2007)

Abbreviations: *adk*, adenylate kinase; *vapD*, virulence‐associated protein D.

After the analysis of the 16S rRNA gene of ORT isolates, the sequence identity of ORT in GenBank has been established through BLAST (Basic Local Alignment Search Tool) analysis.

#### 2.3.2 Phylogenic analysis

The evolutionary history was inferred using the Neighbour‐Joining method (Saitou & Nei, 1987). The percentage of replicate trees in which the associated taxa clustered together in the bootstrap test (1000 replicates) is shown next to the branches (Felsenstein, 1985). The evolutionary distances were computed using the Kimura 2‐parameter method (Kimura, 1980) and are in the units of the number of base substitutions per site. This analysis involved 15 nucleotide sequences. All positions containing gaps and missing data were eliminated (complete deletion option). There were a total of 740 positions in the final data set. Evolutionary analyses were conducted in MEGA X (Kumar et al., 2018).

#### 2.3.3 Detection of virulence genes in ORT

ORT isolates were analysed to determine the *adk*, *vapD* and *copA* genes. The PCR reaction for *adk*, *vapD* and *copA* at a final volume of 15 µL comprised: 7.5 µL of Master Mix, 1 µL of each primer (10 pmol/µL), 5 µL of template DNA and 0.5 µL of sterile distilled water. The thermal cycle conditions and forward and reverse primers for *adk*, *vapD copA* genes are stated in Table 1.

PCR products of 10 µL were stained by electrophoresis (90 V for 1 h) in 1% agarose gel with 0.5 µg/mL safe stain, and DNA fragments were observed by UV transillumination and compared with a 100 bp DNA marker. Serotype A of ORT and distilled water were used as positive and negative controls, respectively.

#### 2.3.4 ERIC and RAPD PCR analysis

The ERIC‐PCR and RAPD‐PCR reactions at a final volume of 25 µL consisted of: 12.5 µL of Master Mix, 2 µL of each primer (10 pmol/µL), 5 µL (500 ng) of template DNA and 5.5 µL of sterile distilled water. The sequences of primers and thermal application for ERIC‐PCR and RAPD‐PCR are stated in Table 1.

ERIC‐PCR and RAPD‐PCR products of 10 µL were stained by electrophoresis (60 V for 3 h) in 2% agarose gel with 0.5 µg/mL safe stain, and DNA fragments were observed by UV transillumination and compared with a 50 bp DNA marker. Distilled water was used as a negative control.

### 2.4 Antibiotic susceptibility

Antibiotic susceptibility testing of ORT isolates was performed by the Kirby‐Bauer disc diffusion method in the M31‐A3 document, according to the Institute of Clinical Standards and Standards Institute (Clinical and Laboratory Standards Institute [CLSI]) tests (CLSI, 2018). A suspension of the 0.5 McFarland of the isolates was prepared and then inoculated to the surface of the Muller Hinton agar medium with 3% horse blood by a sterile cotton swab and tested with 25 different disks of antimicrobial agents (PadtanTeb Co): gentamicin 10 µg (GEN), tetracycline 30 µg (TE), neomycin 30 µg (N), linco‐spectin 109 µg (LS), tylosin 30 µg (TY), tilmicosin 30 µg (TMS), trimethoprim/sulfamethox 23.7 µg (SXT), amikacin 30 µg (AN), nalidixic acid 30 µg (NA), erythromycin 15 µg (ERY), ceftriaxone 30 µg (CRO), ampicillin 10 µg (AM), nitrofurantoin 300 µg (FM), furazolidone 100 µg (FX), streptomycin 10 µg (S), penicillin 10 µg (P), cefazolin 30 µg (CZ), cefixime 5 µg (CFM), cefoxitin 30 µg (FOX), cloxacillin 1 µg (CX), enrofloxacin 5 µg (NFX), fluorfenicol 30 µg (FF), fosfomycin 200 µg (FOM) and soltrim 25 µg (SL). The diameter of the growth inhibitory areas was measured, and the results were compared according to standard tables (CLSI, 2018).

### 2.5 Data analysis

The data were analysed by SPSS 16.0 statistical software. The chi‐square test and Fisher's exact test were used to analyse the data. The ERIC and RAPD PCR images were uploaded to NTsys software for analysis. The data obtained from Excel software were entered as 0 and 1, depending on the presence or absence of bands. Respectively, the Jacquard similarity coefficient between each dendrogram of isolates was also calculated by the NTsys‐pc programme, and these data were analysed by the UPGMA cluster by the NTsys SAHN programme.

## 3 RESULTS

### 3.1 Identification

The results of culture and biochemical experiments showed that 23 (11.5%) isolates from turkey cleft palate swabs were identified as ORT. After 24 h of incubation in the blood agar medium, grey to white‐grey colonies were observed, and after 48 h of incubation, the diameter of the colonies increased significantly. Moreover, the isolates showed no growth on McConkey agar. Only two isolates (8.7%) were beta‐haemolytic, and the other 21 (91.3%) isolates were non‐haemolytic. Gram staining revealed rod‐shaped gram‐negative microorganisms and pleomorphic. The isolates were oxidase positive and negative for catalase, urease, gelatinase and indole. They fermented glucose, sucrose, maltose, lactose and arabinose but could not ferment sorbitol.

### 3.2 PCR confirmation and partial 16S rRNA ORT gene sequence analysis

**FIGURE 1:**
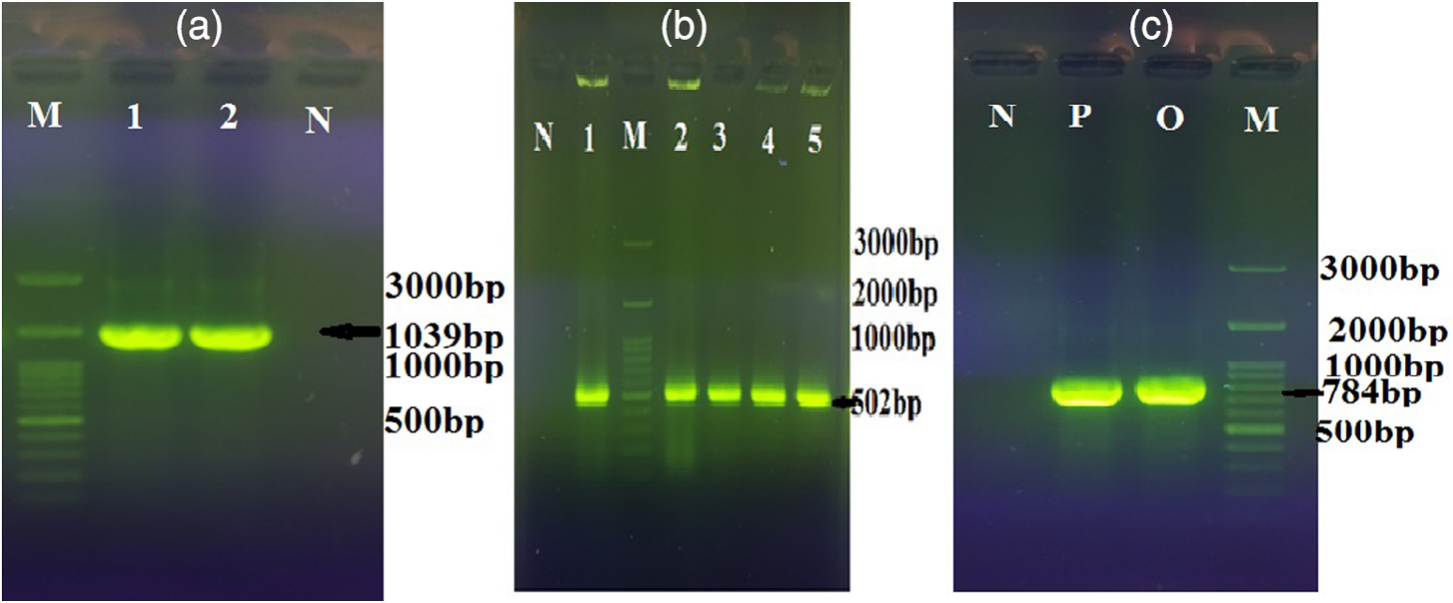
Electrophoresis of PCR products. (a) *copA* gene with the 1039 bp length (Lane M: 100 bp DNA marker, Lanes 1 positive control, Lane 2: positive *copA* gene isolate, Lane N negative control). (b) adenylate kinase (*adk*) gene with the 502 bp length (Lane N: negative control, Lanes 1–4: positive *adk* gene isolates, Lane M: 100 bp DNA marker and lane 5 positive control) and (c) 16S rRNA specific band of ORT isolates with 784 bp length (Lane N negative control, Lane P positive control, Lanes O ORT positive isolate and Lanes M: 100 bp DNA marker).

According to molecular characteristics, all 23(100%) ORT isolates were positive in the PCR assay and produced an amplified product of 784 bp (Figure 1).

### 3.3 Phylogenic analysis

**FIGURE 2:**
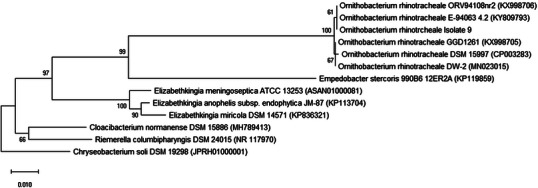
Neighbour‐joining phylogenetic tree based on 16S rRNA gene sequences shows the position of *Ornithobacterium rhinotracheale* isolate nine among closely related species. *Chryseobacterium soli* DSM 192,988 was used as an out group. Bootstrap values are shown as percentages of 1000 replicates; only values above 60% are shown. Bar: 0.01 substitutions per nucleotide position.

Phylogenic analysis of the 16S rRNA sequence of ORT isolates showed 100% similarity with reference strains isolated in France and the Netherlands. It also has less than 100% similarity with the reference strains isolated in the United Kingdom, Germany and China where all the isolates were isolated from lung and nasal swab turkey (Figure 2).

### 3.4 Virulence genes distribution

All 23 (100%) isolates had the *adk* gene, but both *copA* and *vapD* genes were present in two (8.7%) isolates and seven (30.43) isolates (Figure 1).

### 3.5 ERIC PCR assay

**FIGURE 3:**
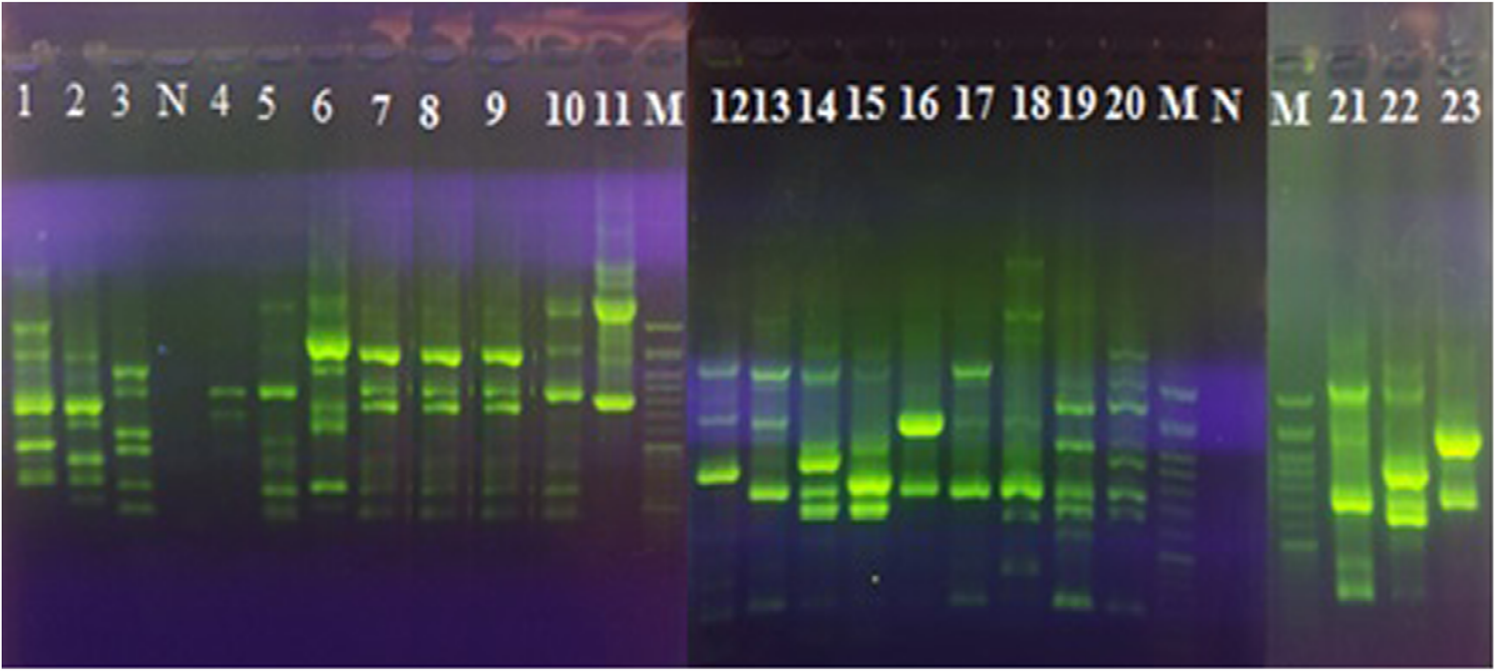
Electrophoresis of enterobacterial repetitive intergenic consensus (ERIC) PCR products of ORT isolates. Lane N: negative control, Lanes M: 100 bp DNA marker and Lanes 1 to 23: The different genetic patterns ORT isolates.

The ERIC‐PCR results showed 20 different patterns, and the isolates were classified into four different clusters based on the ERIC‐PCR results (Figure 3). The dendrogram image obtained from the cluster analysis shows that the highest ERIC genotype cluster from the ORT profiles produced was found in A cluster (comprising 10 isolates), followed by two clusters B and C (consisting of 5 isolates) and cluster D (comprising 3 isolates). Clusters A and B had isolates with less genetic distance than each other, and isolates in cluster D had more genetic distance.

### 3.6 RAPD PCR assay

**FIGURE 4:**
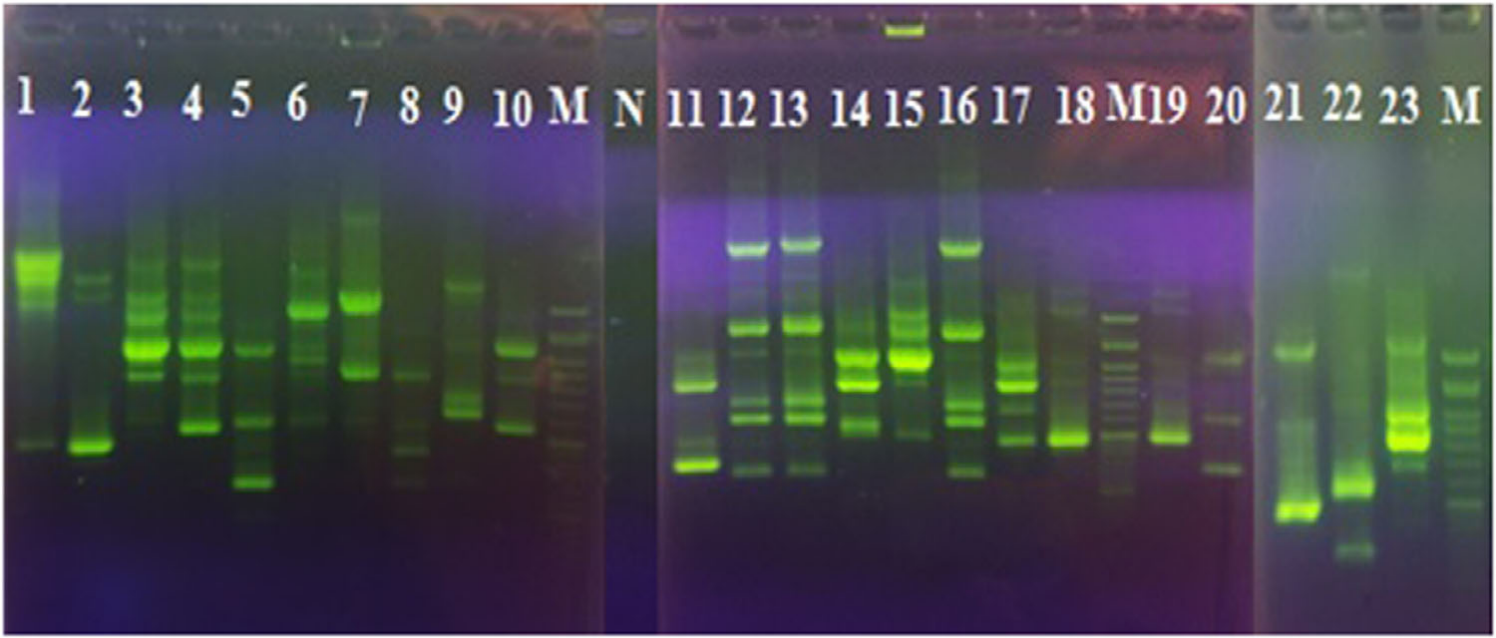
Electrophoresis of RAPD PCR products of ORT isolates. Lane N: negative control, Lanes M: 100 bp DNA marker and Lanes 1 to 23: The different genetic patterns ORT isolates.

Twenty‐one different patterns of isolates were also identified based on RAPD‐PCR results (Figure 4). Moreover, the dendrogram images obtained from the gene cluster analysis in the RAPD‐PCR showed four distinct gene clusters. The highest RAPD genotype cluster from the ORT profiles produced was found in the C cluster (consisting of 8 isolates), followed by two clusters A and D (consisting of 6 isolates) and cluster B (containing 3 isolates). Isolates in cluster C have higher genetic diversity and are at a greater genetic distance than other isolates.

**FIGURE 5:**
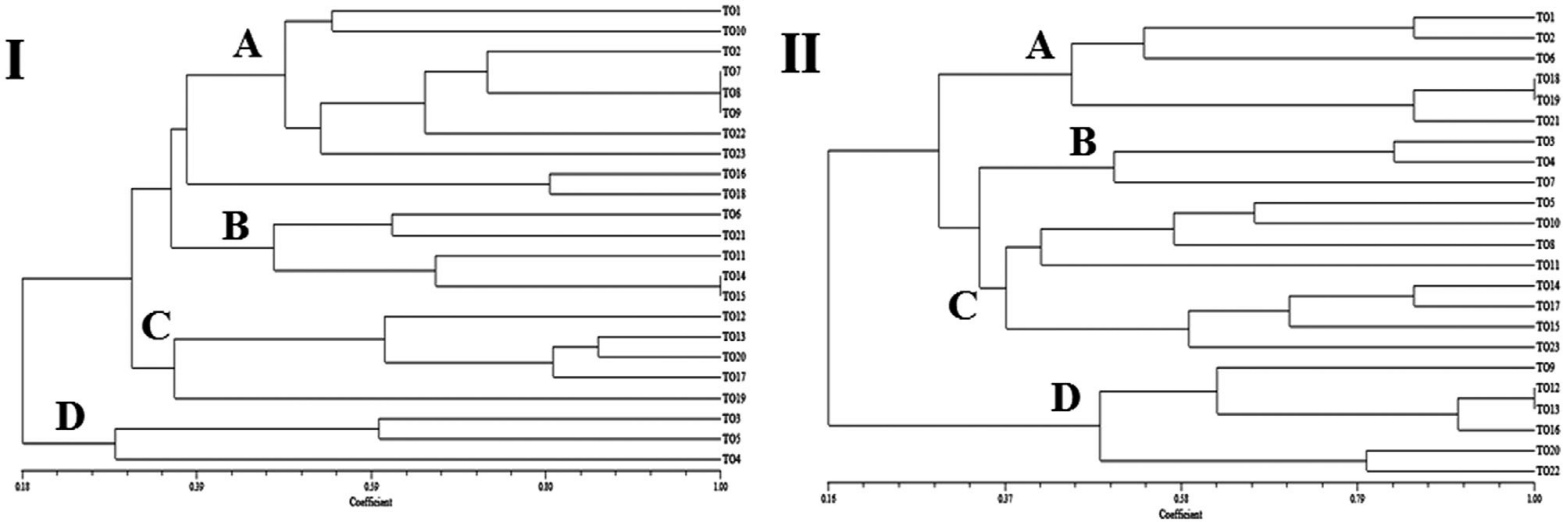
(I) Dendrogram grouping the *Ornithobacterium rhinotracheale* (ORT) isolates based on the enterobacterial repetitive intergenic consensus (ERIC)‐PCR results with the ERIC 1 primer. Cluster analysis and similarity values between fingerprints were based on the Dice coefficient and UPGMA. All 23 strains were grouped into four genetic clusters (A to B). (II) Dendrogram grouping the ORT isolates based on the RAPD assay with the M13 primer. Cluster analysis and similarity values between fingerprints were based on the Dice coefficient and UPGMA. All 23 strains were grouped into four genetic clusters (A to B).

Some ORT strains showed that there is no relationship between ORT pathogenic genes and fingerprint profiles based on ERIC‐PCR and RAPD‐PCR dendrograms (Figure 5).

### 3.7 Antibiotic susceptibility

The antibiotic susceptibility results showed that all isolates (100%) were sensitive to tetracycline, ceftriaxone, tylosin, tilmicosin, cefoxitin, cefixime, soltrim, linco‐spectin, fluorophenicol and furazolidone, and most isolates (78.26%) were resistant to gentamicin, amikacin, cefazolin, streptomycin and penicillin. All isolates (100%) were resistant to cloxacillin and fosfomycin. No correlation was found between the presence of virulence genes and antibiotic resistance (Fisher's exact and chi‐square test: *p* > 0.05). Moreover, there was no significant difference between antibiotic resistance and the data obtained from ERIC‐PCR and RAPD‐PCR assay (chi‐square test: *p* > 0.05).

## 4 DISCUSSION

Respiratory diseases have been reported as one of the major problems of the poultry industry worldwide. ORT is known as the most important cause of this disease. The prevalence and spread of this bacterium differ among bird species. As the clinical symptoms caused by ORT are of little importance because of their similarity with other respiratory diseases; therefore, diagnosis of infection based on isolation and molecular identification of ORT is necessary to control and treat the disease (De la Rosa‐Ramos et al., 2018).

In the present study, for the isolation of ORT, no antibiotics were used in the blood agar medium to isolate both antibiotic‐sensitive and antibiotic‐resistant ORTs. In various studies, 10 µg of gentamicin was added to the blood agar medium containing 5%–10% sheep blood. The isolation of ORT is more successful by adding gentamicin to the culture medium. However, it cannot isolate more than half of the ORT‐sensitive in samples; therefore, further research is needed to isolate the ORT from the mixed bacterial population (Smith, 2021).

Studies have reported that biochemical methods for detecting ORT are not specific enough (Hassan et al., 2020). Therefore, because of their specificity and sensitivity, molecular techniques are used in the definitive diagnosis of ORT infections (Umali et al., 2018). In Iran, the prevalence and spread of ORT infection in broiler flocks in different parts of the country have been studied. However, studies on the isolation and identification of ORT in turkeys are few. Asadpour et al. (2011), in their study, showed that out of 290 trachea swab samples from 29 broiler flocks slaughtered in Gilan, Iran, three samples were positive for ORT by culture and PCR methods. In 2021, Karimi Dehkordi et al. examined samples of the lung, trachea, airway, infraorbital sinus, hook joint, heart blood, brain, spleen, intestine and kidney of 30 turkeys in Isfahan. PCR results showed that 53% of the turkeys were infected with ORT. Moreover, their study showed that although ORT is found mainly in the respiratory tract, it can be systemic and infect some other organs, including the joints, brain, liver, spleen and heart blood, but it cannot affect the intestines and kidneys (Karimi‐Dehkordi et al., 2021). Mirzaie and Hassanzadeh (2013) reported the prevalence of *Ornithobacterium* by PCR in turkeys, partridges, quails and pigeons. Asadi et al. (2022) also reported that out of 60 swab samples from the trachea of 42 broiler and 18 broiler breeder flocks, 13 isolates were positive for *Ornithobacterium*. Doosti et al. (2011) showed that out of 375 samples of tracheal swabs and lung tissue of turkeys slaughtered in Isfahan, Iran, 75 samples (19.93%) were positive for ORT by PCR. Many studies have been performed on the isolation and identification of ORTs worldwide. In 2020, Nisar et al. examined the molecular detection of 60 bacterial isolates suspected of ORT from chicken and turkey in California and Minnesota. Fifty‐six of the isolates were confirmed as ORT by PCR of the 16S rRNA gene. The results of the genetic diversity of isolates using MLST showed that there was less genetic diversity in California isolates (Nisar et al., 2020). A study by Hasan et al. in Egypt examined 180 samples from broiler trachea, lungs and air sacs for ORT. A higher percentage of ORT infection was found in lung samples than in trachea and air sac samples (Hassan et al., 2020). Hassan et al. reported for the first time in Iraq that out of 67 broiler chickens with swollen head syndrome, 28 samples were positive for ORT (Al‐Hasan et al., 2021). This study showed the prevalence of ORT in commercial poultry flocks. A study by Umali et al. in eastern Japan examined four laying hen farms with mild to intense respiratory disease and decreased egg production and feed intake. Three of these four farms were ORT positive. ORTs of 21 were isolated from 65 sampled birds (Umali et al., 2018). Churria et al., In Argentina, showed that out of 82 swab samples from broiler trachea and lung tissue, 50 were identified as ORT‐positive samples (Gornatti‐Churria et al., 2019; Umali et al., 2018).

Determining the genetic relationship of different isolates can help to find the source of infections and the distribution of specific clusters in various geographical areas. ERIC PCR and RAPD PCR are important because of their ability to differentiate between different ORT isolates, and ERIC‐PCR has been used in most studies to differentiate closely related bacterial species (Thachil et al., 2007). Amonsin et al. (1997) used ERIC‐PCR to investigate 54 ORT strains isolated from poultry in various countries between 1983 and 1995. They determined the isolates had seven distinct fingerprint patterns that did not match their geographical origin. Strains isolated from chickens and turkeys were indistinguishable by this method, although the ERIC fingerprints of isolates recovered from wild birds differed from those of poultry samples (Amonsin et al., 1997). In another study, Koga and Zavaleta (2005) analysed 25 ORT isolates from chickens by ERIC‐PCR, and all isolates produced the same pattern. Waldow (2009) found a correlation between serotype and RAPD pattern by M13 universal primer. In the study of Szabo et al., they identified 13 different ERIC patterns from 37 isolates obtained from commercial birds. In their research, RAPD PCR assay was performed with OPG11, OPH19 and M13 primers, and 10 different patterns of isolates were identified with the M13 primer. The other two primers were not suitable for the identification and grouping of isolates. They also reported that ERIC‐PCR was the most discriminating method to investigate the genetic diversity of ORT isolates (Szabó et al., 2017). In the study of Vargas et al., 9 reference strains and 23 isolates of ORT from diseased respiratory poultry from Mexico were serotyped and genotyped. Seven distinguishable ERIC genotypes were detected among the nine reference strains of ORT included in the study. All 23 Mexican isolates of ORT in the study shared a unique ERIC genotype (Peña‐Vargas et al., 2016). Tachil et al. obtained 10 different fingerprints with M13 and 6 different fingerprints with ERIC PCR from 58 isolates of ORT. They reported that the M13 fingerprinting technique was found to be more discriminative in differentiating ORT isolates than the ERIC fingerprinting technique. They suggest that fingerprinting techniques may be a more discerning tool for characterizing ORT isolates (Thachil et al., 2007). In this research, the isolates showed 20 different genetic patterns based on ERIC PCR results and were classified into four different clusters. Based on the results of RAPD PCR ‘with M13 primer’, 21 different genetic patterns were observed. According to the dendrogram images obtained from gene cluster analysis in ERIC‐PCR, clusters A and B had isolates with less genetic distance than each other, and isolates in cluster D had more genetic distance. Moreover, the dendrogram images obtained from gene cluster analysis in RAPD‐PCR showed that the isolates in cluster C have higher genetic diversity and are located at a greater genetic distance than other isolates. Although the RAPD method showed the greatest genetic diversity, however, it is recommended to use both ERIC and RAPD methods because both ways are simple and inexpensive and the showed genetic diversity of isolates well. Considering the diversity in ORT strains from the studied turkeys, there is a possibility that this organism has a relatively long evolutionary history in this population. As the nucleotide sequences of the strains in this study showed significant similarity to strains from France, the Netherlands, China and Germany (Figure 2), it is possible that the evolutionary origin of the circulating strains in the studied turkey population is from these countries and perhaps due to migratory wild birds. Proving this hypothesis requires further research.

Studies are needed to identify virulence factors and invasion pathways. This will enable a better understanding of the pathogenic mechanism of this bacterium. In addition, they will be needed to control ORT infections and minimize the economic cost and losses of poultry farms (De la Rosa‐Ramos et al., 2018). The presence of neuraminidase and haemagglutinin in ORT has been described. These virulence factors may play a role in host colonization and inflammation. Although haemolysin is a virulence factor in ORT, recent studies show that non‐haemolytic isolates can survive longer in the host and have higher pathogenicity. The mechanism of ORT adhesion in the host tissue is unknown, but haemagglutinin and other glycoproteins may play a role in this process (Kastelic et al., 2013; Walters et al., 2014; Zahra et al., 2013).

In previous studies, pORI plasmid carrying possible virulence factors, heavy metal resistance genes and other sequences associated with potential proteins in ORT have been reported (Jansen et al., 2004; Smith et al., 2020; Smith, 2021). In the present study, two haemolytic isolates were identified, and these two isolates contained the virulence genes *adk* and *vapD*, which may be related to their pathogenicity. In addition, two isolates contained the *copA* gene, and seven isolates contained the *vapD* gene, which indicates a plasmid in these isolates (Smith et al., 2020). In the study of Erfan et al., which aimed to investigate the virulence factors of bacteria causing respiratory diseases, the *adk* gene was identified as the virulence factor in ORT, and Thieme et al. also showed the *adk* gene in ORT (Erfan and Marouf, 2019; Thieme, Hafez, et al., 2016; Thieme, Mühldorfer, et al., 2016). In the present study, the *adk* gene was investigated by PCR, and all isolates carried this gene. The results were consistent with previous studies.

One of the most significant issues in the poultry industry that should be considered is the different antibiotic resistance patterns and the emergence of multidrug resistance in ORT isolates. Although autogenic vaccines are commonly used, effective antibiotics are essential in the event of a disease outbreak. In this research, all ORT isolates were sensitive to tetracycline, ceftriaxone, tylosin, tilmicosin, cefoxitin, cefixime, soltrim, linco‐spectin, fluorophenicol and furazolidone. Most isolates were resistant to gentamicin, amikacin, cefazolin, cloxacillin, streptomycin, penicillin and fosfomycin. No correlation was found between the presence of virulence genes and antibiotic resistance (Fisher's exact and chi‐square test: *p* > 0.05). Moreover, there was no significant difference between antibiotic resistance and the data obtained from ERIC‐PCR and RAPD‐PCR assay (chi‐square test: *p* > 0.05). In a study by Mayahi et al. (2016) on 210 tracheal swab samples from 21 broiler flocks slaughtered in abattoirs, it was reported that all isolates (100%) were sensitive to tetracycline, florfenicol and cephalexin, and 89% of the isolates were resistant to phosphomycin, soltrim and gentamicin. Hassan et al. reported the resistance of most isolates to amoxicillin, cephradine, gentamicin and levofloxacin and their sensitivity to colistin and doxycycline. All the isolates were moderately sensitive to erythromycin, neomycin, trimethoprim and tetracycline (Hassan et al., 2020). According to Asadi et al. (2022), many ORT isolates were resistant to tetracycline, doxycycline, ciprofloxacin and ceftizoxime, whereas 12 isolates were sensitive to furazolidone. Vargas et al. found that 9 reference strains and a total of 23 isolates of *ORT* from respiratory diseased poultry in Mexico were resistant to ampicillin, colistin, fosfomycin, gentamicin, kanamycin, penicillin, streptomycin and trimethoprim‐sulfamethoxazole, and 20 of the 23 isolates were susceptible to amoxicillin and clavulanic acid (Peña‐Vargas et al., 2016).

## 5 CONCLUSION

Considering the prevalence of ornithobacteriosis infection in chicken and turkey flocks, quick diagnosis of infection with molecular methods and the use of an antibiogram before treatment with common antibiotics are necessary.

The results of the present study showed the prevalence of ORT in turkey with a high level of resistance to some common antibiotics. ORT was isolated by culture and confirmed by PCR in this study. It seems that if all suspected samples were analysed by PCR, the prevalence would be higher. Moreover, both ERIC and RAPD PCR are distinctive methods for investigating the genetic diversity of ORT isolates. These data may be helpful for the typing of isolates in epidemiological studies and serve as the foundation for designing region‐specific vaccines for future use.

## AUTHOR CONTRIBUTIONS

*Methodology; investigation*: Sedigheh Yusefinejad. *Supervision; conceptualization; methodology; formal analysis; writing – original draft; writing – review and editing*: Darioush Gharibi. *Supervision; methodology; investigation*: Mohammad Khosravi. *Methodology*: Mansour Mayahi and Masoud Reza Seyfi Abad Shapouri. All authors read and approved the final manuscript.

## CONFLICT OF INTEREST STATEMENT

The authors declare that they have no known conflicts of interest or personal relationships that could have appeared to influence the work reported in this paper.

### ETHICS STATEMENT

The ethical approval was not required for the present study as the samples were collected from the slaughtered turkey.

### PEER REVIEW

The peer review history for this article is available at https://www.webofscience.com/api/gateway/wos/peer‐review/10.1002/vms3.1490.

## Data Availability

Data available on request from the authors.
